# Methodology for Energy Optimization in Wastewater Treatment Plants. Phase III: Implementation of an Integral Control System for the Aeration Stage in the Biological Process of Activated Sludge and the Membrane Biological Reactor

**DOI:** 10.3390/s20154342

**Published:** 2020-08-04

**Authors:** Ana Belén Lozano Avilés, Francisco Del Cerro Velázquez, Mercedes Lloréns Pascual del Riquelme

**Affiliations:** 1 Water Department, VECTORIS, S.L., Espinardo, 30100 Murcia, Spain; 2 Department of Electromagnetism and Electronics, Faculty of Chemistry, Campus of Espinardo, 5, Espinardo, 30100 Murcia, Spain; fcerro@um.es; 3 Department of Chemical Engineering, Faculty of Chemistry, Campus of Espinardo, 5, Espinardo, 30100 Murcia, Spain; llorens@um.es

**Keywords:** wastewater treatment plant, energy optimization, control system, management strategy

## Abstract

The proposed methodology for optimizing energy efficiency, based on good management of the aeration process through the implementation of an appropriate control strategy, achieved reductions of more than 40% in energy consumption at the San Pedro del Pinatar Wastewater Treatment Plant (WWTP) (Murcia, Spain). Phases I and II of this methodology managed to reduce the oxygen needs of the microorganisms in the biological system, optimize the efficiency of oxygen transfer to the biological reactor and redesign the installation to correct abnormal energy loss situations. In addition, we established the basis for Phase III, which implemented a control strategy to achieve stable values close to the setpoints of the fundamental operating parameters of the aeration process. The control system is based on the measurements recorded by strategically installed sensors and mathematical algorithms based on models, achieving an expert adaptive-predictive system that regulates aeration both in the biological stage by activated sludge and the aeration of the installed ultrafiltration membrane system. The objectives were: (i) to achieve automatic execution of the best management strategy; (ii) to reduce the energy demand; (iii) to improve the operation and stability of the process; (iv) to reduce operating costs; and (v) to contribute to the fulfillment of the sustainable development objectives.

## 1. Introduction

In recent years, the increase in population and the requirements of new regulations for the quality and treatment of wastewater, in compliance with European Directive 91/271/EEC [1], has considerably increased the number of wastewater treatment plants and the interest in expanding and modernizing old facilities. This has led to an increase in energy consumption in the wastewater treatment sector [2]. In Spain, each Autonomous Community has a general plan for sanitation and treatment; in the case of the Region of Murcia, the last plan developed covers the period from 2015 to 2025 [3].

As far as urban wastewater is concerned, the treatment processes are essentially of a biological nature, with the aeration stage of the microbiological bed representing the greatest energy consumption. Therefore, special attention must be paid to the aeration stage when optimizing the energy of the WWTP.

The cost of energy at the San Pedro del Pinatar wastewater treatment plant in Murcia, Spain, built to treat a flow of 20,000 m^3^/day of wastewater, represents more than one-third of the operating costs [4]. This WWTP incorporates a biological treatment system consisting of an activated sludge biological reactor (AS) and an ultrafiltration membrane biological reactor (MBR). Therefore, an aeration system is required for the purification activity of the bacteria. Another one is required to control the fouling of the membranes and to guarantee the proper functioning of the system, which results in high energy consumption.

Approximately 68% of energy consumption in the WWTP comes from biological treatment by AS, and 53% of consumption is associated with the operation of the MBR membrane biological reactor [4]. One of the main disadvantages of MBR systems for wastewater treatment is their high operational cost compared to conventional activated sludge technology. One of the main challenges in this type of installations [5,6,7,8] is to achieve a reduction in operating costs. For this reason, a methodology is proposed to achieve energy optimization of the biological aeration stage in the WWTP, which estimates the potential savings that can be achieved in the WWTP, based on continuous monitoring and control of the parameters that characterize the process.

The first phase of this energy optimization methodology consisted of adjusting the concentration of suspended solids in the liquor mixture in the biological reactor to minimize the oxygen requirements of the microorganisms [4]. The second phase was based on optimizing the operating conditions of the air injection systems and redesigning the installation so that the supply of the necessary oxygen was made as efficiently as possible and the air supply requirements were reduced [9]. The third and final phase of optimization, which is the subject of this article, was the implementation of a new control strategy with the main objective of minimizing the deviations between the target set values and the actual values of the parameters to be controlled, as set in previous phases. This translates into a reduction in equipment operating times and, consequently, a reduction in energy consumption. In addition, the specific objectives were to:

- Ensure the correct functioning of the different components involved in the operation of the WWTP, maximize the performance of the available equipment, coordinate the operation of the different process units in order to get the most out of them and reduce the impact of the disturbances on the final recipients of the treated water.
- Guarantee the quality of the effluent.
- Save energy by automatic control and by using the tariff system, by making possible the displacement of part of the energy consumption in aeration to the most economic tariffs.
- Contribute to achieving the energy targets set for 2030, at local and national level, by the United Nations for sustainable development [10], to comply with the European Energy Strategy for 2020 and for 2030 [11,12].

## 2. Initial Operating Conditions of the Control Systems Regulating the Biological Aeration Stage

The proposed methodology for optimizing energy efficiency, based on the selection of good management of the aeration process and its achievement over time through the implementation of an appropriate control strategy, achieved reductions of more than 40% in energy consumption at the San Pedro del Pinatar Wastewater Treatment Plant (WWTP) (Murcia, Spain). Phases I and II of this methodology managed to reduce the oxygen needs of the microorganisms in the biological system, optimize the efficiency of oxygen transfer to the biological reactor and redesign the installation to correct abnormal energy loss situations. In addition, we established the basis on which we developed Phase III, which consisted of implementing a control strategy capable of achieving stable values close to the setpoints of the fundamental operating parameters of the aeration process. The control system is based on the measurements recorded by strategically installed sensors in the AS biological reactor and in the MBR and mathematical algorithms based on models, achieving an expert adaptive-predictive system that regulates both aeration in the biological stage by activated sludge and aeration of the installed ultrafiltration membrane system. The objectives were: (i) to achieve the automatic execution of the best management strategy; (ii) to reduce the energy demand; (iii) to improve the operation and stability of the process; (iv) to reduce the operation costs; (v) to contribute to the fulfillment of the sustainable development objectives; and (vi) to represent the previous step for the digitalization of the installation. Pedro del Pinatar WWTP consists of two plug flow reactors with prolonged activated sludge aeration, with a volume per reactor of 8015 m^3^. Each reactor is divided into four zones, a first anoxic chamber followed by three oxic chambers with a decreasing number of diffusers, 696, 408 and 300 units, respectively, for a total of 1404 diffusers per reactor. The movement of the mixed liquor was carried out by means of submerged, wide-bladed agitators, which provide the minimum agitation during the period of low loads, when the specific contribution of air is lower than the minimum advisable.

On the other hand, the air supply to the oxidation channels was carried out by six rotary piston blowers with a 110 kW motor, with a flow of 3165 Nm^3^/h at 6 m.w.c. Two of the blowers have an electronic frequency variator and the other four have an all-or-nothing operation. For air distribution in the biological reactor the installation had fine bubble diffusers with a diameter of 9. A general aeration pipe led the pressurized air through the blowers to the biological reactors. The air flow was distributed to each of the reactors through a line equipped with two automatic regulation valves, and to the different aerobic chambers through the manual regulation of three valves installed in each of the branches. The regulation of the air flow for each of the reactors was carried out by means of a control loop programmed in the WWTP’s Programmable Logic Controller (PLC), based on the measurement of dissolved oxygen provided by a single probe installed in the No. 3 oxygen chamber. It was completed by the pressure signal from the general collector and the working pressure setpoint, which sought to minimize the supply pressure and thus the associated energy consumption.

**Figure 1:**
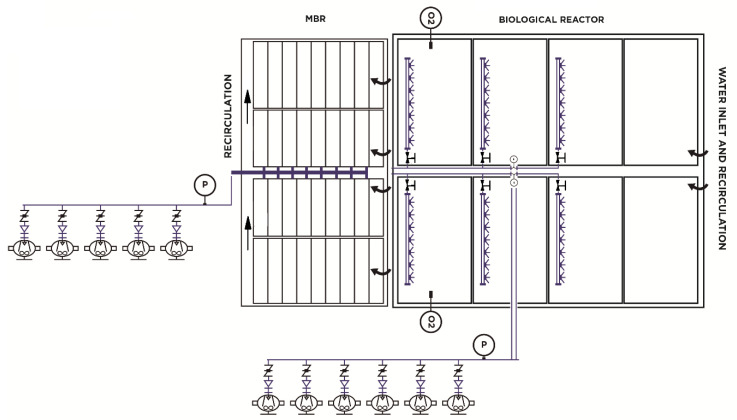
Initial process flow diagram of aeration operation.

The biological degradation process was completed with a membrane treatment system composed of four trains with eight modules/membrane train, made of polyvinyl difluoride hollow fiber, with 1516 m^2^ of installed membrane surface, which provided an excellent quality effluent without the need for subsequent water disinfection. The membrane biological reactor consisted of five trilobular rotary piston blowers with a 75-kW motor, which provided a maximum instantaneous air flow per cassette of 425 Nm^3^/h and a minimum flow per cassette of 272 Nm^3^/h. This was sufficient for cleaning the membrane surface and did not have a variable speed drive. A diagram of the initial aeration system is shown in Figure 1.

For process control, the San Pedro del Pinatar WWTP used classic on/off and proportional, integral and derivative (PID) methods [13]. When trying to control a WWTP that had complex and variable dynamics over time, with controls of this type, reacting to the error already produced, the result was unstable and not satisfactory in regulating the critical variables of the process, which were generally difficult to control. Such control was useful in many cases, but in others its performance was inadequate and usually required adjustments by experienced operators.

This type of control became even more complicated when trying to control the activated sludge process and the dynamics involved in the nutrient removal processes taking place in the biological reactor. In addition to being complex reactions that vary over time, they have process parameters that can change due to temperature and load variations in the influent, etc. This lack of rigorous control led to significant variations in the variables to be controlled with significant energy losses [14].

The average loads and flows received since the start-up of the San Pedro del Pinatar WWTP were much lower than those foreseen in the design, with high hydraulic retention times (over two days). This contributed to the high energy consumption, although it allowed us to modify the usual way of working for this type of reactor, based on a continuous and prolonged aeration of the mixed liquor contained in the oxic chambers with an internal recirculation depending on the amount of nitrates to be eliminated [15,16,17], by other possible ways:

➢ Operation with time-based start-stop aeration cycles, regardless of the oxygen values achieved
➢ Cycle operation with start-stop aeration according to pre-established oxygen settings in oxic chamber No. 3, regardless of the real oxygen requirements of the system

In both modes of operation, the water was passed through the reactor in alternating aerated and anoxic-anaerobic cycles. This achieved an acceptable performance in the removal of nutrients (nitrogen and phosphorus compounds) with significant energy savings compared to conventional plug flow operation.

This control strategy presented great limitations as it required the process operator to select, based on his knowledge and experience, the duration of the aeration cycles. Alternatively, the process operator had to select the oxygen concentration setpoints to be achieved in the aerated areas and the value of the pressure in the air line required to achieve the oxygen concentration at the measurement point (oxic chamber No. 3). For complex and time-varying processes, this form of control would result in an excess of aeration to guarantee the process performance or in legal violations due to failures in nitrogen removal. In addition, since the system did not have ammonium/nitrate measurement probes, the response to any type of deviation was postponed until a laboratory result was obtained [18,19,20].

For this reason, the need to optimize this mode of operation was detected, and it was necessary to implement a new control system based on the continuous measurement of the water quality parameter to be controlled, the pressure in the collector and the measurement of oxygen in each of the oxic zones in the biological reactor. To achieve effective nutrient removal, the oxygen concentration in the oxic zones should be high enough to allow the growth of appropriate organisms to ensure nitrification and to maintain the mixture. At the same time, it should also be low enough to avoid excessive agitation and to reduce, as far as possible, the dissolved oxygen in the anoxic zones induced by nitrate recirculation. This form of aeration regulation could lead to significant savings in the energy bill of the plant [21,22,23,24].

On the other hand, the aeration of the MBR system consisted of four membrane trains for the separation of the effluent from the biological sludge by cross-flow filtration, so that the cake was removed from the membrane surface by means of an agitation flow. This distribution of trains caused substantial differences in the fouling of the membranes, with the degree of clogging being greater in the modules closest to the entrance of the train and less in those further away.

**Figure 2:**
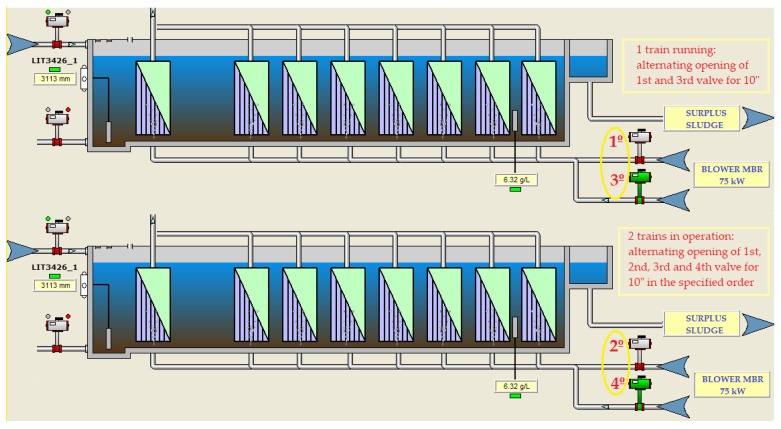
Actuation sequence of the aeration control valves for one or two membrane trains in operation.

The agitation flow for cleaning was caused by the continuous bubbling of air under the membranes, so that the ascension of the bubbles provided the agitation and an extra supply of oxygen. The air blowing process was very inefficient as the air flow was through perforated tubes and the air inlet was in the opposite direction to the mixed liquor inlet to the trains. Moreover, this air supply was made in a sectorized way through a cyclic and timed on/off control of the air inlet valves to each of the membrane trains. This made it possible for the air to go to either train when two trains shared the same aeration blower:

- For water flows below 300 m^3^/h, one membrane train with a 75-kW aeration blower was in operation. This blower provided a unit air flow of approximately 3650 Nm^3^/h at 3.72 m.w.c. distributed through two cyclic valves with a 10”/10” actuation mode. One of the valves would be open for 10 s, ventilating one part of the membrane cassette, and closed for 10 s, ventilating the other part of the cassette by alternating operation of the other valve, thus uninterruptedly (Figure 2).
- For water flows of 300–600 m^3^/h, two membrane trains and a 75-kW aeration unit were in operation. The air distribution was done by four cyclic valves with a 10”/30” aeration mode. These valves would act sequentially; they would open for 10 s and remain closed for 30 s, with the actuation sequence being repeated indefinitely while the trains were in operation (Figure 2).
- For water flows of 600–900 m^3^/h, three membrane trains would come into operation, and, for flows of 900–1200 m^3^/h, four membrane trains would be required to be in operation. In both cases, two 75-kW blowers were operated following the same valve actuation sequence mentioned above for one or two trains.

This way of operating meant a limitation in the energy saving when the flow demand to be treated by the membranes required an odd number of membrane trains. This meant a large energy cost when working between 30% and 55% of the time in this disadvantageous condition, depending on the season. In addition, the uninterrupted supply of oxygen to the MBR system was not used in the process of organic matter degradation and nutrient removal and was limited to an air supply to agitate the membrane fibers and to prevent contamination.

Therefore, it was essential to replace the classic strategy of aeration control by an adaptive-predictive expert control system combining the instantaneous values provided by the installed sensors, which measure the performance of the biological process and the control parameters of the membrane fouling [25,26], with mathematical models that allow predicting and adapting the form of control to the reality of the installation.

## 3. Method for the Design of the Aeration Control System

**Figure 3:**
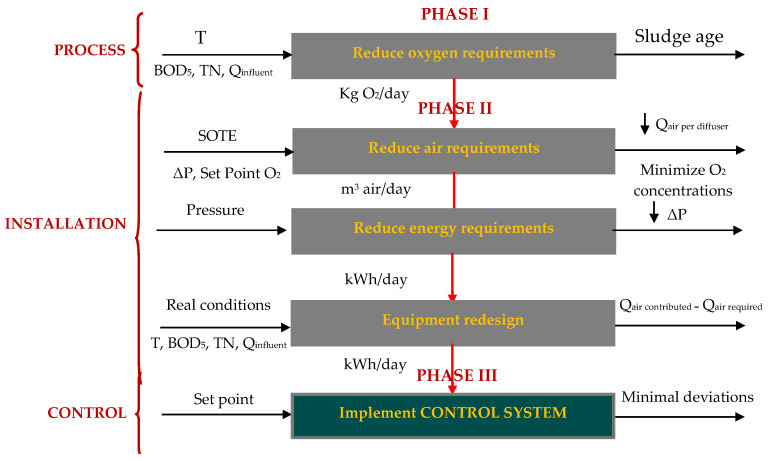
Stages included in an energy optimization of an aeration system.

The San Pedro del Pinatar WWTP started from a fairly high average specific consumption value, around 1.03 kWh/m^3^. This was largely associated with the aeration of the biological reactors, the pumping and stirring of active sludge and the aeration of the bioreactor membranes necessary to prevent their contamination. This value of specific energy consumption was reduced by around 0.67 kWh/m^3^ after the first two phases of optimization of the proposed methodology [8,9] (Figure 3). In the first phase of optimization (Phase I), the oxygen requirements of the microorganisms in the biological system were reduced. The process variables that became important were the flow values (Q_influent_), pollutant load received, expressed in kg/day of the biological oxygen demand (BOD_5_), total nitrogen (TN), temperature (T) and sludge age. In Phase II, we optimized the efficiency of the oxygen transfer (SOTE) to the biological reactor and redesigned the installation to correct abnormal situations of energy loss or pressure drop in the air line (ΔP) and over-supply of air flow to the biological reactor (Q_air per diffuser_).

To complete the energy optimization of the WWTP, Phase III was developed to improve the control strategy that regulated the biological aeration stages for the elimination of nutrients and for the control of membrane fouling, as mentioned above. In this way, a methodology for energy optimization of the WWTP was completed, the phases of which are provided in Figure 3.

### 3.1. Design of the Aeration Control System of the Activated Sludge Biological Reactor

**Figure 4:**
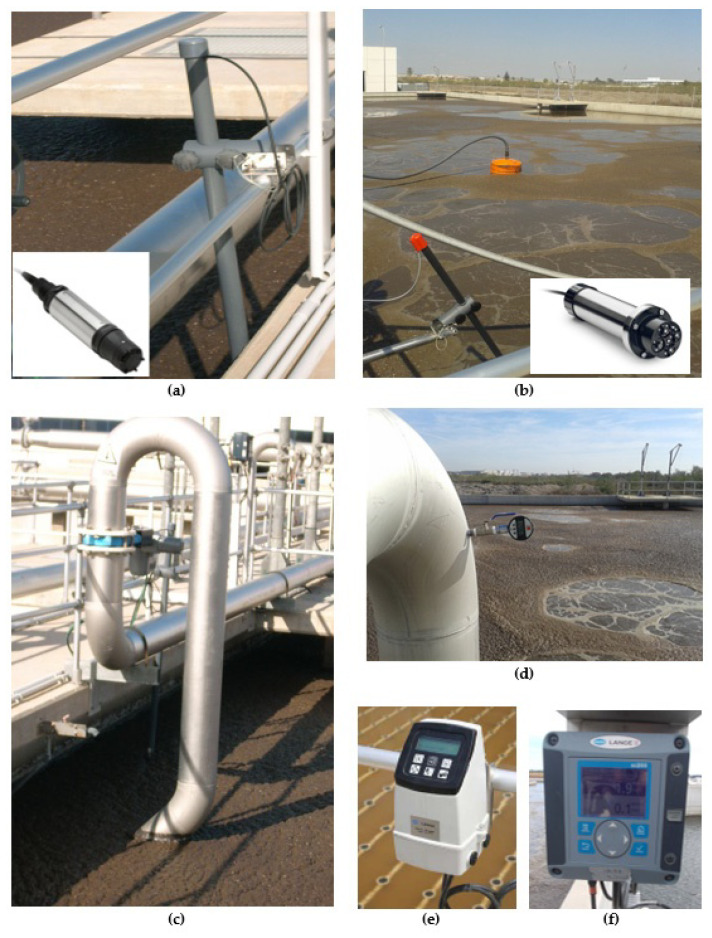
(**a**) Dissolved oxygen sensor; (**b**) combined ammonium and nitrate sensor; (**c**) motorized airline valve; (**d**) pressure transducer; and (**e**,**f**) dissolved oxygen and ammonium/nitrate controllers, respectively.

To optimize the operating mode of the activated sludge biological aeration system, a new control system was implemented based on the continuous measurement of ammonium in the biological reactor, pressure in the air manifold and the measurement of oxygen in each of the aerated zones. This was implemented both in the AS biological reactor and in the MBR, providing greater flexibility and continuity in the response and exceeding the human factor of control [27,28,29,30]. The control strategy developed is based on the installation of sensors for the measurement of fundamental parameters in the process and mathematical algorithms of probability calculations and fuzzy logic techniques. These used the time profiles as inputs, obtained from monitoring of the parameters (ammonium and dissolved oxygen), to achieve the appropriate levels of purification and process control with minimum energy cost [31,32,33,34]. To carry this out, the following actions were necessary (Figure 4):

- Installation of a sensor for continuous measurement of ammonium and nitrate in each line of the reactor, located at the end of oxic chamber No. 3, formed by a combined sensor for continuous measurement, by selective electrode, of the ammonium and nitrate ions, with a measurement range of 0.5–1000 mg/L NH_4_^+^-N and 0.5–1000 mg/L NO_3_-N.
- Installation of additional sensors for the measurement of dissolved oxygen (DO) in oxic chamber Nos. 1 and 2 of both biological reactors. This, together with the sensors in oxic chamber No. 3, allowed the dissolved oxygen concentration to be determined by luminescence in each of the chambers, with a measurement range of 0–20 mg/L.
- Installation of a dissolved oxygen (DO) sensor in the MBR, specifically in the sludge recirculation channel common to all the membrane trains. This made it possible to determine the dissolved oxygen concentration by luminescence in the mixed liquor, with a measurement range of 0–20 mg/L O_2_/0–50 °C.
- Installation of motorized guillotine valves in each downpipe to regulate the air flow in the oxic chambers with variable dimensions depending on the density of diffusers in each chamber with WAFER connections, and a cast iron body. An AUMA MATIC electric actuator was also installed, with regulation by means of a 0–20-mA signal.
- Installation of pressure sensors in each airline to control the pressure, with a measurement range of 0–1 bar.
- Programming in the PLC of the new control loop according to the measured parameters (NH_4_^+^-N, NO_3_^−^-N and DO) and the pressure in the manifold, as well as implementation in the plant Supervisory Control and Data Acquisition (SCADA) system.

**Figure 5:**
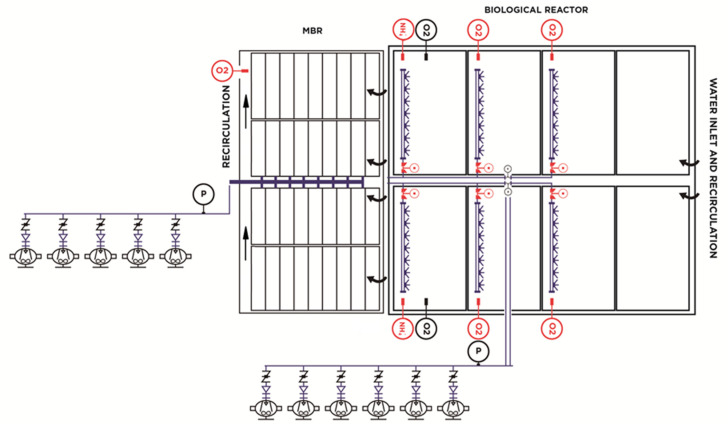
New aeration operation scheme.

**Figure 6:**
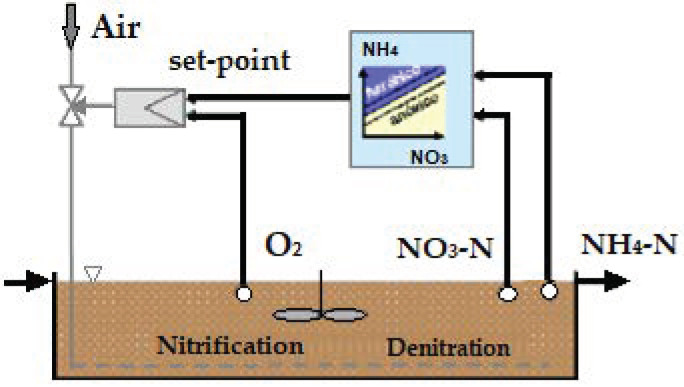
New aeration operation scheme.

The new scheme of the aeration system is shown in Figure 5. The control scheme is shown Figure 6 and described in the following sections.

#### 3.1.1. Criteria for Establishing Aeration Cycles Based on the N-NH_4_^+^/N-NO_3_^−^ Content in the Water

As a starting point, an initial outlet setpoint value was established for the ammonium concentration, taking into account compliance with the water quality always required by the contract (instantaneous value < 10 mg/L NH4^+^-N). This setpoint was modified to maximize the elimination of nutrients and minimize the associated energy consumption, based on the daily analytical results obtained in the laboratory. This setpoint was defined once the operation of the WWTP began, based on the new control strategy [35,36,37,38,39].

The ammonium concentration in the oxic chamber No. 3 marked the starts and stops of the aeration system, the starting frequency being the minimum required (33 Hz). The stop cycles of the system were limited by a time “**t**” in minutes, which can be configured from the SCADA system, to avoid excessive sludge sedimentation. Similarly, the time that the aeration equipment remained in operation was limited to a maximum time “**T**” in minutes, which can be configured from the SCADA system and was independent of the recorded value of NH_4_^+^/NO_3_^−^ to avoid excessive aeration due to faults in the reading of the probes.

The possibility of automating the stops of the blowers by measuring the nitrates provided by the probe was also studied, but it was ruled out due to the existence of interference in the measurements of this parameter due to the chloride content of the water.

#### 3.1.2. Criteria for Establishing Oxygen Set Points in Aerated Areas

The oxygen setpoints were established based on the average NH_4_^+^/NO_3_^−^ values obtained at the reactor outlet. The system allowed different oxygen setpoints to be set in each of the aerated zones to optimize oxygen transfer into the reactor [2,40,41,42]. Oxygen transfer depended largely on the concentration gradient between the oxygen saturation value and the concentration in the reactor itself according to Equation (1). This meant that the lower is the working concentration in the reactor, the higher is the transfer [9]. Therefore, the criteria used to establish the oxygen setpoints in each chamber were to set lower working setpoints in the head-end aerated areas, since it was in this area that the highest oxygen demands and the lowest alpha values (α) or transfer coefficient [43,44,45,46] coincided.

(1) $$\mathrm{TTO}=K_{L}*\frac{A}{V}*(C_{S}-C_{L})=K_{L}*\alpha *(C_{S}-C_{L})$$

where TTO is the oxygen transfer rate in mg/L/h, K_L_ is the mass transfer coefficient in the liquid phase, C_S_ is the concentration of dissolved oxygen in the reactor, C_L_ is the oxygen saturation concentration under field conditions and V is the volume of liquid that has a total interfacial area A in contact with the gas phase. The A/V ratio is the specific surface area, usually represented by α [47].

The start of the control process involves establishing initial values as a setpoint for the oxygen concentration to be achieved in each of the chambers, values that vary over time depending on the results obtained in NH_4_^+^/NO_3_^−^ in the biological reactor output current. To obtain the desired value in the parameter to be controlled, the control system has implemented a slope to increase the setpoint, which facilitates reaching the desired values and working most of the time at the lowest possible operating frequencies of the blower, seeking the minimum energy consumption.

In addition, to achieve a greater adjustment in energy saving, the aeration control system was designed to be able to work year-round with different oxygen set points according to three time bands with different electricity rates (off-peak hours 1:00–8:00 a.m., peak hours 9:00–16:00 and flat hours 17:00–24:00). The aim was to adapt, as far as possible, the hourly flow demand curves to the energy price curve which varies according to the time bands of the day.

The function of the dissolved oxygen measurement probe in the membrane chamber is limited to a check of the DO concentration in the final stage. This makes it possible to reduce the oxygen setpoints to be fixed in the oxic zones of the biological reactor by AS, by exploiting the oxygen transferred to the bioreactor membranes [48,49,50]. A discrepancy in the reading obtained from DO in the MBR causes an alarm message to be sent for early verification and correction.

#### 3.1.3. Criteria for Establishing Working Pressure Setpoints

Implementation in the plant SCADA system of the different control strategies of the aeration system of the WWTP studied, based on the working pressure, allowed selection, at each moment, of the most suitable strategy. It also allowed the configuration of parameters from the different control screens that were integrated and built with the logic of the local system. Two cases are distinguished:

1. Tests with constant pressure settings.

In this operating mode, a constant pressure setpoint value was set that was sufficient to facilitate the control of the oxygen levels in the reactors, setting action times on the increase/decrease of the blower operating frequency to reach the pressure setpoint.

2. Tests with variable pressure set points.

In this case, initially, the maximum opening and closing degree of the air regulation valves located in each of the branches was defined by a maximum pressure value, which coincided with the system pressure with all the air regulation valves open and the blower working at full capacity (50 Hz). It was also defined by a minimum regulation pressure value that coincided with the minimum air flow specified in the performance curves of the diffusers and the minimum working flow provided by the blower. Initially, the regulation range of the valves was set between 50% and 80%, a configurable value to achieve effective and fast regulation for this WWTP.

The variation of the working pressure is made according to the degree of opening of the valves and the oxygen levels measured at each moment in the system and its deviation from the set point. Modifications are made to increase or decrease the set point pressure according to the situations developed [51,52]:

- When in one of the zones, the control system requests that the position of the corresponding valve in one of the chambers is above the maximum opening threshold set (80%, value configurable from the SCADA system) as the oxygen setpoint is not reached in the zone and is lower than the setpoint by a certain value (setpoint + 0.01). This situation is maintained for a certain time (5 min), and then the setpoint of the PID pressure controller is increased by a certain amount (0.002 bar). The resulting increase in pressure in the main line has the effect of increasing the measured oxygen value in the tank in question, bringing it back to the setpoint. This reduces the opening of the corresponding valve, positioning it in a more desirable range for control purposes.
- If, on the other hand, the control system requires the position of the corresponding valve in one of the chambers to be below the minimum opening threshold set (50%, value configurable from the SCADA system), because the oxygen measurement is higher than its setpoint by a certain value (setpoint−0.01), and this situation is maintained for a certain time (5 min), the PID pressure controller setpoint is decreased by a certain value (0.002 bar). The resulting decrease in pressure in the main line has the effect of decreasing the measured oxygen value in the area in question, reducing it to its set point, and increasing the opening of the corresponding valve, positioning it in a more desirable range for control purposes.

The controller’s setpoints and the time that elapsed between each new decision were made modifiable from the SCADA system, as were the pressure increments at each regulation step. These two parameters were the most important to control.

**Figure 7:**
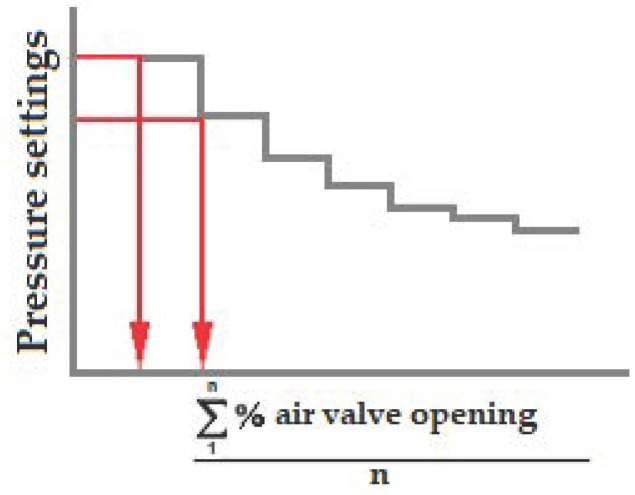
Graph to determine the degrees of opening of the air valves.

To increase the speed of the control response variants of this strategy were studied, in which we replaced the control response based on increases in working pressure as a function of the opening of the control valve and the oxygen levels in the system, with the calculation of working pressure increases as a function of the degree of valve opening calculated as an average value. These experiences had been previously developed in other WWTPs in the Region of Murcia (Spain) [53,54]. Using the same calculation basis and the data obtained in our tests, the first point was to look for the relationship between the working pressure settings and the average values of percentage of valve openings, as shown in Figure 7.

With the desired average opening degree calculated from the results in Figure 7 and taking into account the actual opening degree of the valves, the desired response speed of the control system and a system-specific proportionality factor, increments were established of the working pressure (ΔSP pressure) calculated in a moment from the following formula:

(2) $$\Delta \mathrm{SPpressure}\;t=[\frac{\sum_{1}^{n}\%\;\mathrm{air}\;\mathrm{valve}\;\mathrm{opening}\;t}{n}-a]\times \left[\frac{\sum_{1}^{n}\%\;\mathrm{air}\;\mathrm{valve}\;\mathrm{opening}\;t}{n}-a\right]{\;}^{1/c}\;]\times b$$

where a is the degree of actual opening of the valves, c is a number inversely proportional to the desired system response speed and b is a proportionality factor specific to each system.

Thus, the control system calculated an increase (positive or negative) in the set pressure, increasing the value of the pressure increase as we move away from the target value, thus achieving a more stable control system that always tended to balance. The pressure set point can be calculated in a time t, from the set point in t − 1, by means of Equation (3).

SPpressure _t_ = SPpressure _t−1_ + ΔSPpressure (3)

### 3.2. Design of the Aeration Control System for the Membrane Bioreactor

As commented in Section 2, the process of aeration in the MBR is very inefficient and assumes a great consumption of energy. This is mainly due to the excess of air that is supplied to the membranes for their agitation and cleaning and its non-use as part of the process of degradation of the organic matter and/or elimination of nutrients in the water.

For this reason, the aeration control strategy was modified, for which the following were necessary:

➢ *Reduce the air flow supplied to the membrane system*.

The installation of an electronic frequency converter (VF) for the motor of one of the 75-kW aeration blowers, made it possible to carry out tests by reducing the flow of air injected by varying progressively from 50 (maximum frequency) to 33 Hz. This represents maximum reductions in flow rate of 34%. The aim was to establish the minimum optimum airflow values without disturbing the operation of the membrane filtration system. A control panel was established in the plant SCADA system for the adjustment of the blower operating speed, allowing, at the operator’s discretion, two ways of working:

- Set a fixed blower operating speed
- Set two different speeds with a settable time so they follow each other cyclically

The control parameters used to establish the limits of reduction in the operating frequency of the aeration equipment, without altering the quality of the filtration process, were the membrane permeability values and the transmembrane pressure values (TMP). These were provided instantaneously by the measurement of the vacuum pressure provided by the vacuum gauges installed in the permeate line.

➢ *Separate the aeration shared between membrane trains.*

**Figure 8:**
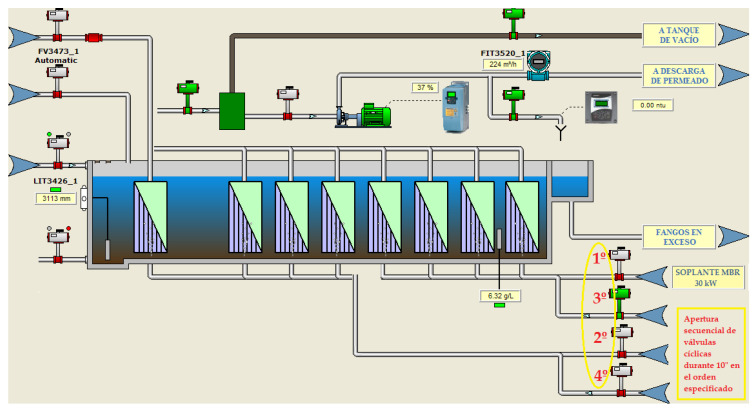
Actuation sequence of the aeration control valves in Single-train mode.

**Figure 9:**
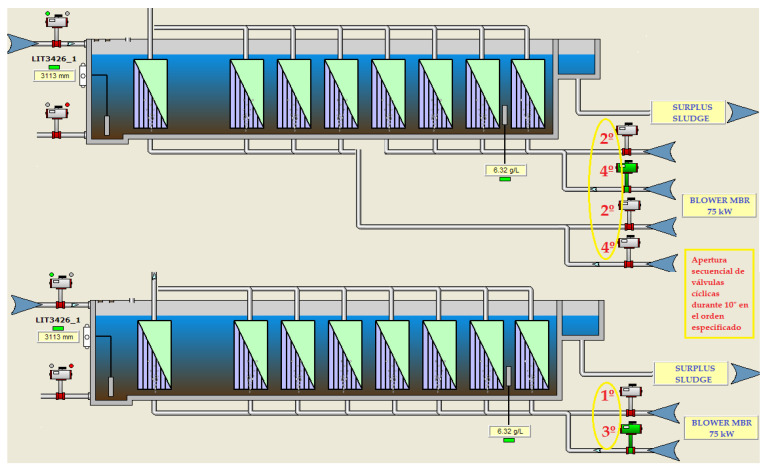
Actuation sequence of the aeration control valves in two-train mode.

To reduce the energy invested in the operation (odd-numbered) of one or three membrane trains, it was necessary to modify both the aeration system and its control philosophy, with the main objective of eliminating excess agitation. The most important actions carried out were:

➢ Reduce the aeration flow rate of one of the installed equipment to 50%.
  To do this, an economic study was carried out, considering the possibilities:
  - Replacing an existing 75-kW aeration unit with a new unit with an installed power of 30 kW, with a normal air flow rate of approximately 1825 Nm^3^/h at 300 mbar.
  - Mechanical modification of the transmission system of one of the existing blowers to achieve the reduction in operating speed required to obtain the desired flow rate, no less than 1825 Nm^3^/h at 300 mbar.
➢ Modify the air supply line to isolate the aeration between trains.
  The intervention was made to two trains, identified as No. 2 and No. 3. For each modified train, it was necessary to install:
  - Two new pneumatically operated cyclic valves with 1/4” BSP connection actuator and a maximum pressure of 8 bar.
  - A new pneumatic panel, where all the electric and pneumatic control elements were included: electrovalves, pressure regulator, etc.
  - A manual butterfly valve type WAFER Butterfly DN-300 and manual reducer to facilitate the maintenance of the cyclic valves.
  - Pressure sensors in each airline to control the pressure, with a measurement range of 0–1 bar.
➢ Program PLC and SCADA with a new control strategy based on the number of trains running:
  - When the demand for water treatment requires the startup of a single train (single-train mode), train No. 2 or train No. 3 is activated according to its availability in the SCADA system with actuation of both the blower with a 30 kW motor and the four cyclic valves located in the same train with 10/30” opening and closing (Figure 8.)
  - When the demand for water treatment requires the start-up of two trains (two-train mode) there is an option to start up trains Nos. 1 and 2 or trains Nos. 3 and 4, depending on their availability in the SCADA with a 75-kW blower motor. The operation criterion followed will be the same as up to now (10/30” opening and closing) but it is necessary to set up four cyclic valves (Figure 9).
  - When the demand for water treatment requires the implementation of three trains, the operation is a combination of the two previous cases, in which a 75-kW blower for two-train operation (two-train mode) with 10”/30” aeration and a 30-kW blower for single-train operation (single-train mode) with 10/30” aeration would come into operation (Figure 8 and Figure 9).
  - When the demand for water treatment requires the operation of four trains, all available trains with two 75-kW blowers and the performance criterion developed for the two-train mode are put into operation (Figure 9).

## 4. Results and Discussion

### 4.1. Design of the Aeration Control System of the Activated Sludge Biological Reactor

**Figure 10:**
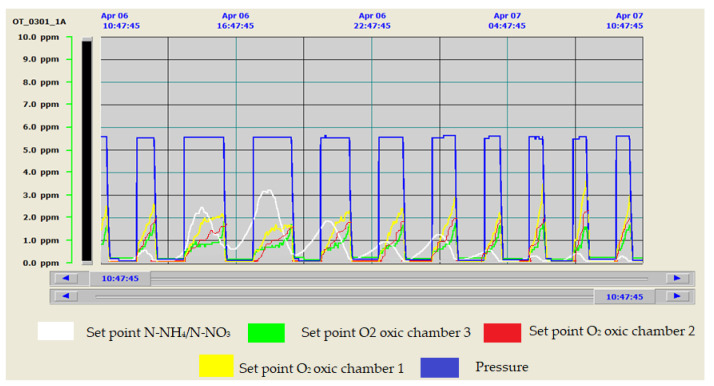
Evolution of Ammonium/Nitrate, oxygen in the three oxygen chambers and working pressure.

The results obtained by the optimization of the biological aeration regulation strategies implemented at the San Pedro del Pinatar WWTP were interesting and in line with other tests carried out at WWTPs in Murcia. For example, in WWTP Ceuti, the energy consumption resulting from the aeration was reduced by 28% with saving of 35% of the overall operating cost of the WWTP [52,53]. These are dynamic control systems that allow adaptation to the different situations that may arise [55,56,57] (Figure 10), ensuring more stable working conditions, saving energy and complying with water quality requirements.

**Figure 11:**
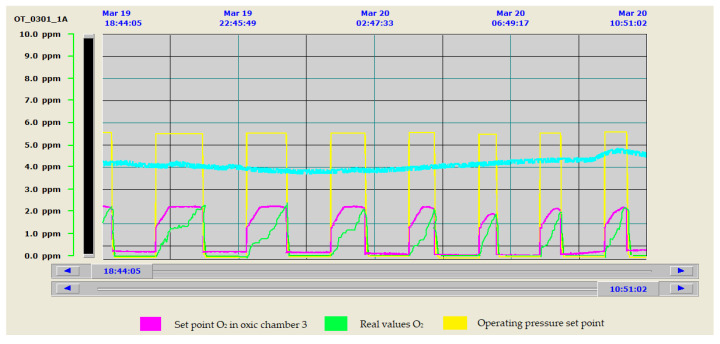
Oxygen evolution in Zone 3 with the old control system.

**Figure 12:**
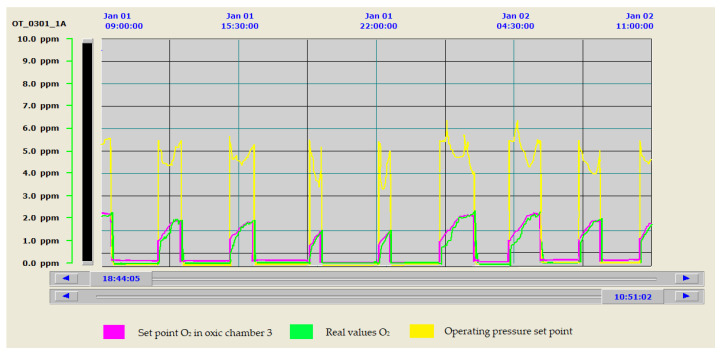
Oxygen evolution in Zone 3 with the new control system.

To evaluate the efficiency and benefit that this type of control represented with respect to the old system, the evolution of the oxygen levels reached in the biological reactor is shown in both cases. The old control strategy is shown in Figure 11 and the new one is shown in Figure 12. As can be seen in Figure 11, when working with the old control system, there are significant discrepancies between the set points and the target values of the control system, resulting in significant process mismatches and energy losses. Figure 12 shows how the control system allows the values of the control parameters to be perfectly adjusted to the selected target values. It is also interesting to see how, with the new control, we were able to adapt the aeration to the lower electricity tariff periods. This reduced the number of hours of blower operation at peak times, aspects not considered in Figure 11, where the shortest aeration cycles occurred in the period of the cheapest tariffs.

Another interesting aspect that can be seen in Figure 11 is that aeration control strategies based on maintaining constant working pressure resulted in higher than necessary working pressure set points for the manifold. These set points were set for the most unfavorable flow and pollution load conditions in the effluent to ensure nutrient removal performance. Reducing the constant pressure setpoint to tighter values sometimes caused deficiencies in the nutrient removal process.

As can be seen in Figure 12, the new configuration of the control system led us to work most of the time at the lowest possible blower operating frequencies. This resulted in improved oxygen transfer and lower air velocities, reducing the pressure drop in the diffusers and pipe network, as already verified in the studies carried out in Phase II of the optimization methodology [9].

In addition, the possibility of working with variable pressure allowed the working pressure of the collector to be regulated and adequately minimized, which is the fundamental variable for guaranteeing minimum electrical energy consumption in the aeration system. Figure 12 shows how the pressure of the collector was adapted to the needs of the system, which considerably improved the energy consumption associated with this stage. This fact required the implementation of all relevant parameters for regulation in the SCADA system and made them modifiable, to be able to stabilize the system.

### 4.2. Design of the Aeration Control System for the Membrane Bioreactor

The results derived from the actions carried out to implement the new control system for the biological aeration of membranes resulted in a direct reduction in the energy consumption associated with this stage. This was achieved by substantially reducing the aeration flow rate of the membranes and adapting it to the speed of fouling of the system. This also meant significant savings in the operating costs of the MBR.

The installation of an electronic speed variator in a membrane blower allowed a reduction of 13% in the air supply to the membrane trains, without observing variations in the permeability and TMP values of the membranes before and after the implementation of the action. Before applying the control, the blower drive was kept at a safe value of 50 Hz. This situation kept the permeability very high and consistent, but with higher energy consumption. The variation in the air flow rate injected for cleaning the membranes, by reducing the operating frequency of the blower from 50 to 33 Hz, indicated 43 Hz as the minimum value. In this case, lower permeability values of the membranes and higher TMP values were recorded, indicating the start of membrane fouling.

The actions carried out to make the aeration of the different ultrafiltration membrane modules independent led to the need to reduce the air supply by almost 50% for water treatment demands that required an odd number of membrane trains. The modification of the transmission system of one of the existing blowers made it possible to reduce the air supply from 3650 Nm^3^/h to approximately 1825 Nm^3^/h at 300 mbar, with a reduction in power consumption of almost 60%. If it is considered that approximately 40% of the MBR operating time is in operation with one or three membrane trains, the energy savings made by achieving independence of aeration between trains have proven to be very interesting.

### 4.3. Overall Results on Energy Consumption at the WWTP

Before beginning the development of tests that make up the methodology for the proposed energy optimization, the average specific energy consumption at the San Pedro del Pinatar WWTP was around 1.03 kWh/m^3^. After the first optimization phase (Phase I), energy consumption decreased to 0.83 kWh/m^3^ [4], and, after the second phase (Phase II), consumption reached about 0.67 kWh/m^3^. The implementation of a new biological aeration control system, as the last proposed phase (Phase III), allowed us to consolidate the improvements to the energy ratio achieved in the previous phases. This also contributed to its achievement over time and further adjusting the improvements achieved in this parameter, placing it at the end of the third phase with values around 0.61 kWh/m^3^.

## 5. Conclusions

The main conclusions that can be drawn from the work developed, on the control strategy implemented, based on independent control of dissolved oxygen and pressure in the air line, for each aeration zone and between reactors, are as follows:

- It offers important operational advantages since it is possible to work with both lines of the reactor. This offers a greater capacity for action in the event of an unforeseen event.
- It increases efficiency in oxygen transfer and minimizes air requirements and therefore energy demand, with the correct selection of oxygen concentration and pressure set points.
- It improves process control and provides stability.
- It ensures values in the WWTP outlet water below 1.5 mg/L NH_4_^+^-N and between 1 and 3 mg/L NO_3_^−^N. Ammonium and nitrate removal performance in the effluent water is considerably higher than that required by current regulations.
- It guarantees energy savings of more than 20% in the aeration process of the AS system, by means of exhaustive control of the nitrification and denitrification processes and by adjusting the higher air demand to the most economical electricity rates. Results are in line with those obtained in Phase II of optimization [9].
- Increased control of the aeration of the MBR system reduces the energy consumption of the plant by more than 9.3%.
- It completes a methodology to optimize operating costs in wastewater treatment plants, compatible with European energy policies that promote energy savings and sustainable development through the 17 Sustainable Development Goals. It allows for the consolidation of reductions of more than 40% in the energy ratio expressed in kWh/m^3^.

If we consider an average energy price of 0.11 €/kWh and an average flow treated at the WWTP of 7000 m^3^/day, the savings in operating costs achieved since the beginning of our work is more than 118,041 €/year and the reduction in CO_2_ emissions is of the order of 1078 kg/year. These figures are very interesting given the current interest in wastewater treatment, the increase in population, the requirements of the new directives that regulate the quality of water for discharge and reuse and the need to comply with the demands of energy saving and emissions to the atmosphere.

- The re-engineering of the processes carried out is based on the principles of the circular economy and represents the previous step to digitalization of the installation.
